# The Terminator—A Blood Test for Early Detection of 50 Cancers

**DOI:** 10.1002/puh2.70379

**Published:** 2026-09-15

**Authors:** Abraham Fessehaye Sium, Don Eliseo Lucero‐Prisno

**Affiliations:** ^1^ Department of Obstetrics and Gynecology St Paul's Hospital Millennium Medical College Addis Ababa Ethiopia; ^2^ Department of Obstetrics and Gynecology King Faisal Hospital Rwanda Africa Health Sciences University Kigali Rwanda; ^3^ Department of Global Health and Development, Faculty of Public Health and Policy London School of Hygiene and Tropical Medicine London UK

**Keywords:** cancer, cancer diagnosis, cancer screening, Galleri test, global health, mortality

## Abstract

Globally, cancer remains the second leading cause of premature death. Although there are existing cancer screening programs for early detection of some of the cancers, screening remains ineffective and at times difficult for the deadliest ones, as a result of which patients present in advanced stages with guarded prognoses. Recently, promising results have been found in the largest interventional study (PATHFINDER2) of Galleri test (a multi‐cancer early detection [MCED] test). In this article, we dive below this headline and discuss its implications for actual clinical care and what it may contribute in addressing cancer as a top public health problem.

## 1 Introduction

Cancer continues to be the second leading cause of death worldwide, including premature death [1, 2]. Among females, breast cancer is the most commonly diagnosed cancer and the leading cause of cancer death, followed by colorectal and lung cancer (for incidence), and vice versa (for mortality); cervical cancer ranks fourth for both incidence and mortality. Lung cancer is the most frequent cancer and the leading cause of cancer death among males, followed by prostate and colorectal cancer (for incidence) and liver and stomach cancer (for mortality) [3]. Cancer screening saves lives, but approximately 70% of cancer deaths come from cancers that do not have standard‐of‐care screening and are typically caught too late [4].

There are already a number of examples of established population screening programs that have been implemented, such as mammography (for breast cancer), cytology and HPV (for cervical cancer), and fecal occult blood testing (for colorectal cancer), whereas others have been implemented in different countries with varying degrees of success (e.g., PSA for prostate cancer, HCV for liver cancer). These existing screening programs require expensive infrastructure and highly trained technical and medical personnel. Hence, compliance with recommended screenings has remained poor, especially among historically and socioeconomically vulnerable populations, such as ethnic/racial minorities, under‐insured or uninsured individuals, or those residing in rural areas with limited access to health care services [5].

## 2 The New Breakthrough

The so‐called multi‐cancer early detection (MCED) test has been recently proposed as an innovative and potentially ground‐breaking strategy for screening a wide range of cancers. This test assesses cancer‐related alterations in circulating genomic analytes [6]. The biotechnology company Grail is one of the diagnostic companies that invested in this direction. The company developed a non‐invasive blood test (Galleri test) that detects a signal of over 50 (51 to be precise) cancers by evaluating DNA fragments (especially circulating cell‐free DNA [cfDNA]) in the bloodstream and complementing these measurements with a machine learning algorithm designed to distinguish between healthy or cancer cell origin [7]. An earlier prospective cohort ‐ PATHFINDER (*n* = 6662)‐ assessed the use of this test in oncology and primary care outpatient clinics at seven United States(US) health networks, including hospitals and community‐based clinics, over one year (December 2019 to December 4, 2020). Though the study demonstrated the feasibility of MCED tests, the statistical results presented significant challenges for real‐world implementation. In the study, 92 participants had a “cancer signal detected,” yet only 35 were confirmed to have cancer. Thus, 62% of positive results were false positives, and the positive predictive value (PPV) was approximately 38%. In practical terms, most individuals receiving a positive result did not have cancer. This has important implications for patient anxiety, downstream investigations, and healthcare costs that are not adequately addressed in the study. The mean general anxiety scores increased in both true‐positive and false‐positive groups upon receiving results. Despite these scores generally returning to baseline by the end of the 12‐month study, the interim period caused measurable psychosocial distress. Most participants with false‐positive results still underwent extensive testing (93% received imaging, and 30% underwent invasive procedures, such as biopsies) to rule out cancer. The cumulative cost of these “workups for nothing” was a primary concern. Moreover, 121 participants were diagnosed with cancer, yet 86 of these had a “no cancer signal detected” result at baseline. Direct sensitivity comparisons are methodologically complex;however, the observations from the study underscored that MCED testing cannot substitute for established, organ‐specific screening modalities [8].

Recently, a glimpse of hopehas arrived from another study, transforming early detection ability level for some common and deadly cancers. The largest interventional study of Galleri test (an MCED test)‐ prospective PATHFINDER 2 ‐enrolled 35,878 participants across the US and Canada in a broad, intended‐use population of adults aged 50 and older with no clinical suspicion of cancer. The study addressed the concerns observed in PATHFINDER study. With a refined GRAIL test version used in the study, follow‐up results show an improved PPV of roughly 43%–61% and a higher specificity of 99.5%. A pre‐specified analysis of the first 25,114 participants with at least 12 months of follow‐up shows that adding Galleri to recommended screenings for breast, cervical, colorectal, and lung cancers led to a more than seven‐fold increase in the number of cancers found within a year. Galleri detected approximately three times as many cancers when added to standard‐of‐care screening for breast, cervical, colorectal, lung, and prostate cancers. Interestingly, approximately three‐quarters of the cancers detected by Galleri do not have standard of care screening options and account for most of cancer deaths that occur globally; these include breast, colorectal, lung, and prostate cancers. The other interesting finding is that more than half (53.5%) of the new cancers detected by Galleri were Stage I or II, which can be treated with a cure intent. A key benefit of Galleri is its ability to predict where in the body the cancer is coming from. The test correctly identified the Cancer Signal Origin (CSO) 92% of the time, leading to efficient diagnostic workups [9].

The materialization of this new piece of evidence in practice seems to have a bright future. There is an ongoing clinical trial (NHS‐Galleri), a randomized controlled trial that will assess how well a Galleri test can reduce the number of late‐stage cancers by helping to find cancers early. The trial has enrolled over 140,000 participants (from the general population of England aged 50–77 years who did not have or were not being investigated for cancer). The trial will help the NHS decide whether to introduce screening using this test across the United Kingdom [10].

## 3 Conclusion

The arrival of recent evidence on early cancer detection—using Galleri test for 50 cancers—demonstrates that MCED testing could be promising adjunct *to targeted screening rather than a transformative replacement for existing programs. It could be p*otentially most appropriate in risk‐enriched populations—pending its predictive value.

## Author Contributions

Abraham Fessehaye Sium and Don Eliseo Lucero‐Prisno III developed the concept of the article and wrote the manuscript. Both authors checked the manuscript against intellectual contents and approved the final version for publication.

## Funding

The authors have nothing to report.

## Ethics Statement

The authors have nothing to report.

## Conflicts of Interest

The authors declare no conflicts of interest.

## Data Availability

The authors have nothing to report.
